# R102G polymorphism of the *C3* gene associated with exudative age-related macular degeneration in a French population

**Published:** 2010-07-15

**Authors:** Jennyfer Zerbib, Florence Richard, Nathalie Puche, Nicolas Leveziel, Salomon Y. Cohen, Jean-François Korobelnik, José Sahel, Arnold Munnich, Josseline Kaplan, Jean-Michel Rozet, Eric H. Souied

**Affiliations:** 1 Department of Ophthalmology, Hopital Intercommunal de Creteil, University Paris 12, Creteil, France; 2 Department of Genetics, INSERM U781, Necker Enfants Malades Hospital, Paris, France; 3 University Lille Nord of France, INSERM, UMR744, Institut Pasteur of Lille, Lille, France; 4 Ophthalmologic Centre of Imaging and Laser, Paris, France; 5 Ophthalmology, CHU de Bordeaux, Université Bordeaux 2, INSERM U897, Bordeaux, France; 6 Ophthalmology, Institut de la Vision, Inserm Pierre et Marie Curie University, Paris, France; 7 Unite Fonctionnelle de Recherche Clinique, Creteil, France

## Abstract

**Purpose:**

Major genetic factors for age-related macular degeneration (AMD) have recently been identified as susceptibility risk factors, underlying the role of the complement pathway in AMD. Our purpose was to analyze the role of the R102G polymorphism of the complement component (*C3)* gene in a French population, in a case-control study.

**Methods:**

A total of 1,080 patients with exudative AMD and 406 controls were recruited and genotyped for Y402H of complement factor H (*CFH),* rs10490924 of age-related maculopathy susceptibility 2 (*ARMS2),* and R102G of the *C3* gene.

**Results:**

The distribution of the R102G genotypes was significantly different in the AMD patients compared to controls (p=0.02). The Odds Ratio compared to C/C individuals was 1.4 (95% CI 1.1–1.8) for C/G individuals and 1.4 (95% CI 0.8–2.4) for G/G individuals. In a dominant model, the adjusted Odds Ratio for carriers of the G allele is 1.4 (95% CI 1.0–1.9; p=0.03).

**Conclusions:**

Our study shows *C3* to be a moderate susceptibility gene for exudative AMD in the French population.

## Introduction

Age-related macular degeneration (AMD) is the most common cause of irreversible vision loss in the elderly population in Europe and the United States [1,2]. Identification of risk factors is of major importance for understanding the origins of the disorder and for establishing strategies to prevent AMD. Risk factors for AMD are both environmental [3-9] and genetic [10-18]. Over the past few years, several single nucleotide polymorphisms (SNPs) have been associated with AMD, including variants in the complement factor H gene (*CFH*) and the *ARMS2/HTRA1* locus age-related maculopathy susceptibility 2 (*ARMS2*) [10-17]. AMD has been also associated with other polymorphisms of the complement pathway, such as the complement factor B gene (*CFB*), the complement component 2 gene (*C2*), and the complement component 3 gene (*C3*) [19-30]. Their involvement in AMD together with the finding that drusen contain inflammation proteins [31], strongly suggest the pathway of inflammation and complement in the pathophysiology of the disease.

R102G corresponding to the “fast” and “slow” electrophoretic phenotypes has been associated with AMD in American, British, Dutch, and German populations [22-29]. Our purpose was to analyze the association between this polymorphism and exudative AMD in a French population.

## Methods

### Patients

A total of 1,080 French AMD patients were recruited in four French retinal centers—the Department of Ophthalmology of Creteil in collaboration with center hospitalier universitaire (CHU) de Bordeaux, the Quinze-Vingts Hospital, and the Centre of Imaging and Laser of Paris—between November 2005 and July 2007. Written informed consent was obtained, as required by the French bioethical legislation and local ethic committee comité de protection des personnes (CPP Henri Mondor), and approved by the Declaration of Helsinki for research involving human subjects.

Inclusion criteria of the AMD patients were (1) women or men aged 55 or older and (2) with exudative AMD in at least one eye, and (3) no association with other retinal disease (e.g., diabetic retinopathy, high myopia, or macular dystrophies). Patients underwent a complete ophthalmologic examination, including best corrected visual acuity measurement, fundus examination, and retinal photographs. Fluorescein angiography (FA; Topcon 50IA camera, Tokyo, Japan) and if needed indocyanine green angiography Heidelberg Retina Angiograph (HRA; Heidelberg Engineering, Heidelberg, Germany) and Optical Coherence Tomography (OCTIII Stratus, Carl Zeiss Meditec, Inc., San Leandro, CA) were performed. During the first visit AMD phenotypes in both eyes were analyzed independently by each investigator (E.H.S. and N.L.) before genetic testing, according to color photographs and fluorescein angiography (FA) at presentation. When investigators disagreed on a particular clinical feature, this patient was excluded from further analysis. A questionnaire about medical history was completed.

### Controls

Controls were also recruited in the group of patients who underwent cataract surgery in our four centers. A total of 406 French women or men over 55 years with a normal fundus examination and normal aspect of fundus photography were also recruited at the Department of Ophthalmology of Creteil, France between 2002 and 2008. Information about their medical history, including smoking, was obtained.

### Genotyping methods

Genomic DNA was extracted immediately or after one night preservation at 4 °C from 10 ml blood leukocytes using the reagents from the Illustra Genomic DNA Extraction kit, BACC2, according to the manufacturer’s protocol (GE Healthcare, Little Chalfont, Buckimghamshire, UK). *CFH* Y402H, *ARMS2* rs10490924, and rs2230199:C>G (*C3*:R102G) SNPs were genotyped by quantitative PCR allelic discrimination using reagents and conditions from Custom Taqman SNP Genotyping Assays (Applied Biosystems, Pleasanton, CA), using ABI 7900HT (Applied Biosystems). Five percent of the population was genotyped for quality control; 100% of these duplicates (C3, CFH and ARMS2 SNPs) are concordant. The call rate of the result was controlled by the Hardy–Weinberg Test. All of the SNPs are in Hardy–Weinberg equilibrium.

### Statistical analysis

The Hardy–Weinberg assumption was assessed by the standard method comparing the observed numbers of subjects in different genotype categories with the expected numbers under Hardy–Weinberg equilibrium for the estimated allele frequency and testing with a Pearson goodness-of-fit chi-square (χ^2^) with one degree of freedom.

The χ^2^ test was used to compare allelic and genotype distributions between cases and controls. Logistic regression models were used to estimate the Odds Ratio (OR) with the 95% confidence interval (95% CI) for AMD risk. A stepwise regression method has been used to select covariates (with p<0.15) of the multiple logistic regression model. The OR was adjusted for age, gender, smoking status, hypercholesterolemia status, and CFH and ARMS2 genotypes. Significance levels were set at p<0.05. Analyses were performed with the SAS software release 9.01 (SAS Institute INC, Cary, NC).

## Results

The population consisted of 1,080 exudative AMD cases and 406 controls. The mean±SD age at AMD diagnosis was 79.0±7.4 years. The nongenetic characteristics of the population are shown in Table 1. Cases were significantly older, were less often men and smokers, but had more often hypertension and hypercholesterolemia than controls. However, on the logistic adjusted model, only age, smoking status, hypercholesterolemia, and gender remain significantly different between the two groups. The genotype distributions of the rs1061170 and rs10490924 SNPs within the *CFH* and *ARMS2*, respectively, are shown in Table 2. The genotypic distributions of the *CFH* Y402H and *ARMS2* SNPs were significantly different between cases and controls (p<0.0001).

**Table 1:** Non-genetic characteristics of the AMD patients and controls.

**Clinical datas**	**Cases**	**Controls**	**p**
n	1080	406	
Age, m(sd), years	79.0 (7.4)	67.8 (7.7)	<0.0001
Men, n(%)	366 (33.9%)	163 (40.2%)	=0.025
Hypertension, n/N (%)	567/1061 (53.4%)	138/404 (34.2%)	<0.0001
Smoking, n/N (%)	416/1079 (38.6%)	180/404 (44.6%)	=0.036
Diabetes, n/N (%)	104/1072 (9.7%)	19/286 (6.6%)	=0.11
Hypercholesterolemia n/N (%)	462/1059 (43.6%)	135/403 (33.5%)	=0.0004
BMI, m(sd)	25.5 (4.3)	25.4 (4.3)	=0.86

Abbreviations: m(sd) represents means (standard deviation). BMI indicates Body Mass Index, kg/m^2^.

**Table 2:** Genotype distributions of Y402H of *CFH* and rs10490924 of *ARMS2* among the AMD patients and controls.

**Genotypes**	**Cases**	**Controls**	**Global p values**	Crude OR CI95%*, p	**Adjusted OR CI95%†, p**
*CFH* Y402H (rs1061170)					
TT	229 (21.2%)	160 (39.4%)	<0.0001	1 (ref)	1 (ref)
TC	551 (51.1)	192 (47.3%)		2.0 [1.6–2.6] p<0.0001	2.3 [1.7–3.2] <0.0001
CC	299 (27.7%)	54 (13.3%)		3.9 [2.7–5.5] p<0.0001	4.5 [2.9–7.0] <0.0001
*ARMS2* (rs10490924)					
GG	339 (31.4%)	253 (63.7%)	<0.0001	1 (ref)	1 (ref)
GT	507 (46.9%)	129 (32.5%)		2.9 [2.3–3.8] <0.0001	3.0 [2.2–4.1] <0.0001
TT	234 (21.7%)	15 (3.8%)		11.6 [6.7–20.1] <0.0001	13.2 [7.2–24.2] <0.0001

Abbreviations: OR represents Odds ratio, CI represents Confidence interval. * Non-adjusted OR. † Adjusted for age, gender, tobacco smoking and hypercholesterolemia.

The genotype distributions of rs2230199 of the *C3* gene are shown in Table 3. The rs2230199 genotype distributions of the *C3* gene were in accordance with the Hardy–Weinberg equilibrium (p=0.87 in controls and p=0.13 in cases). The distribution of the rs2230199 genotypes was significantly different in the AMD patients compared to controls (p=0.02). Individuals carrying the G allele were at an increased risk of AMD (OR_G/C_=1.4 CI 95% 1.1–1.8; and OR_G/G_=1.4 CI 95% 0.8–2.4). Adjusted ORs are shown in Table 4. ORs were adjusted for age, gender, tobacco smoking, hypercholesterolemia, and *CFH* and *ARMS2* genotypes. In a dominant model, carriers of the G allele have an adjusted OR of 1.4 (CI 95% 1.0–1.9, p=0.03). In an additive model (the genotype was encoded as 0, 1, or 2, according to the number of risk alleles), the ORs are: crude OR=1.3 [1.1–1.6]/allele, p=0.0084 and adjusted OR=1.3 [1.1–1.7]/allele, p=0.04.

**Table 3:** Genotype distributions of the rs2230199 of the *C3* gene among AMD patients and controls

**Genotypes**	**Cases**	**Controls**	**Global p values**	OR [CI95%]*, p
n	1080	406		
*C3* R102G (rs2230199)				
CC	583 (54.0%)	252 (62.1%)	0.02	1 (ref)
CG	434 (40.2%)	135 (33.2%)		1.4 [1.1–1.8] p=0.008
GG	63 (5.8%)	19 (4.7%)		1.4 [0.8–2.4] p=0.19
G allelic frequency	0.26	0.21	0.009	

OR: Odds ratio CI: Confidence interval * Non-adjusted OR.

**Table 4:** Adjusted OR for age, *CFH, ARMS2* and *C3* gene among AMD patients and controls.

**Age and genotypes**	**OR [CI95%]**	**p OR**	**p global**
Age	1.19 [1.16–1.22]	< 0.0001	
*CFH* Y402H (rs1061170)			
TT	1 (ref)		<0.0001
TC	2.4 [1.7–3.4]	<0.0001	
CC	4.8 [3.0–7.6]	<0.0001	
*ARMS2 *(rs10490924)			
GG	1 (ref)		<0.0001
GT	3.1 [1.2–4.3]	<0.0001	
TT	12.9 [7.0–23.8]	<0.0001	
*C3* R102G (rs2230199)			
CC	1 (ref) *		0.096
CG	1.4 [1.0–2.0] *	<0.04	
GG	1.4 [0.7–2.8] *	<0.33	

Abbreviations: OR represents Odds ratio, CI represents Confidence interval. * Adjusted for age, gender, tobacco smoking, hypercholesterolemia, CFH, and ARMS2 genotypes.

The minor allele frequency was significantly higher in AMD patients than in controls (p=0.009).

## Discussion

Here we report the replication of an association between R102G of the *C3* gene and exudative AMD in the French population. The study of rs2230199 R102G instead of other polymorphisms of the gene (i.e., rs1047286:C>T, C3:L314P) was based on a previous study reporting rs2230199 strongly associated with AMD and on its functional role. This polymorphism is reported to have a functional consequence on the C3 protein. The R102G polymorphism generates the “fast” and “slow” electrophoretic allotypes of C3 (C3F and C3S) [32], showing a differential capacity to bind monocyte-complement receptor C3F [33], which is the risk variant for AMD, and has been previously reported as associated with other immune-mediated conditions, such as IgA nephropathy [34], systemic vasculitis [35], or membranoproliferative glomerulonephritis type II [36].

Prior reports of an association with AMD and R102G of the *C3* gene have been made in American, British, Dutch, and German populations [22-29]. All published studies about C3 and AMD demonstrate a significant association (Table 5). In our study the homozygous individuals carrying the at-risk allele have a lower risk compared to previous publications. Homozygous GG individuals were not significantly associated with a higher risk of AMD compared to the heterozygous GC individuals. The GG genotype was present in only 19 controls, and our findings could be explained by a lack of statistical power, despite the large series of exudative AMD patients tested. It is notable that our sample sizes are a little unbalanced (1,080 cases, 406 controls), which may cause a large variation and power reduction. Another limitation of the study is the significant differences between cases and control populations (Table 1). It remains possible that demographic differences between the group of patients and controls might affect the OR observed in our study. However, logistic regression analyses were performed to adjust for selected covariates.

**Table 5:** Minor Allele Frequencies and OR of the rs2230199 of the *C3* gene in the different studies.

**Studies**	**Number of controls/cases**	**MAF controls**	**MAF cases**	**p**	**OR (CI95%)** **CG individuals**	**OR (CI95%)** **GG individuals**
Yates et al. [22] English Subjects Scottish Subjects Combined Groups	350/603 351/505	0.2 0.2	0.28 0.27	5.9x10^−5^ 5.2x10^−4^*	1.6 (1.2–2.2) 1.8 (1.2–2.6) 1.7 (1.3–2.1)	2.4 (1.3–4.4) 2.9 (1.4–5.9) 2.6 (1.6–4.1)
Maller et al. [24]	934/1238	0.21	0.31	4.51x10^−12^	1.61	3.26
Spencer et al. [23] Case-controls data set Family-based data set	286/701 223	0.21	0.29 0.3	0.001* 0.66	1.55 (1.09–2.21) ‡	
Bergeron-Sawitzke et al. [25]	215/421				1.9 (1.3–2.7)	2.5 (1.1–5.9)
Francis et al. [28]† CEIMDC AREDS	187/211 322/672				0.65 (0.22–1.94) 1.87 (1.38–2.53)	1.93 (1.2–3.1) 3.91 (1.94–7.88)
Despriet et al. [27] Case-control study Rotterdam study Pooled data: Meta-analysis	173/357 4055/476 early AMD 4055/106 late AMD	0.206	0.237 early AMD 0.254 late AMD		1.27 (1.09–1.49) 1.27 (0.9–1.78) 1.46 (1.11–1.92) 1.93 (1.14–3.28) 1.61 (1.46–1.78)	
Park et al. [26] Mayo subjects AREDS subjects Pooled data: Early AMD GA CNV Late AMD (GA+CNV)	299/439 300/1241				1.4 (1.15–1.69) 1.9 (1.5–2.39) 1.8 (1.47–2.22) 1.8 (1.52–2.21)	
Scholl et al. [29] GA	612/99	0.175	0.263	0.0032	1.45 (0.92–2.30)	4.17 (1.67–10.40)
Zerbib et al. [18]	406/1080	0.21	0.26	0.009	1.4 (1.1–1.8) 1.4 (0.8–2.4) 1.4 (1.0–1.9)¨	

Abbreviations: OR represents Odds ratio, CI represents Confidence interval, MAF represents Minor Allele Frequency. OR are for the comparison with the CC genotype. *In All AMD. †CEIMDC Casey Eye Institute Macular Degeneration Center, FBAT Family based association test, AREDS Age-Related Eye Disease Study cohort: categories 4/5 compared with category 1. ‡ represents in additive model, ¨ represents in dominant model.

We enrolled exudative forms of AMD because patients with neovascular AMD are most often referred to our specialized retina departments than atrophic forms of AMD or early and intermediate AMD. The R102G of *C3* was associated with different types of AMD. Yates et al. [22] also showed a significant association with choroidal neovascularization; for case subjects with only geographic atrophy, the association was significant in the English group (p=4.6×10^−4^) but not in the Scottish group, which had fewer subjects with geographic atrophy. In contrast Maller et al. [24] reported no difference in association between exudative AMD and geographic atrophy compared to controls. Interestingly, Park et al. [26] on their pooled data sets on Mayo and Age-Related Eye Disease Study (AREDS) subjects suggest that R102G of *C3* preferentially associates with advanced AMD subtypes than early AMD. Francis et al. [28] revealed in the AREDS cohort that R102G is significantly associated with the progression from early AMD to advanced AMD, with no predisposition to geographic atrophy or neovascular AMD. Scholl et al. [29], analyzing progression of geographic atrophy and the *CFH, ARMS2,* and *C3* variants, did not report an association with the progression of the disease and these variants. Additional studies in prospective cohorts are needed.

In conclusion, our findings reveal *C3* to be a moderate susceptibility gene for exudative AMD in the French population and evidence that the complement pathway has a key role in AMD. Identification and the knowledge of the risk factors and the interactions between all these factors in individuals may allow new therapeutic methods.
